# Viral and Antibody Kinetics, and Mosquito Infectivity of an Imported Case of Zika Fever Due to Asian Genotype (American Strain) in Singapore

**DOI:** 10.3390/v10010044

**Published:** 2018-01-18

**Authors:** Cheong Huat Tan, Li Kiang Tan, Hapuarachchige Chanditha Hapuarachchi, Yee Ling Lai, Pei Sze Jeslyn Wong, Grace Yap, Keng Wai Mak, Wing Yan Wong, Yee Sin Leo, Mei Chun Wong, Lee Ching Ng

**Affiliations:** 1 Environmental Health Institute, Singapore 138667, Singapore; Tan_cheong_huat@nea.gov.sg (C.H.T.); Tan_li_kiang@nea.gov.sg (L.K.T.); Chanditha_Hapuarachchi@nea.gov.sg (H.C.H.); Lai_Yee_Ling@nea.gov.sg (Y.L.L.); Jeslyn_Wong@nea.gov.sg (P.S.J.W.); Grace_Yap@nea.gov.sg (G.Y.); Mak_Keng_Wai@nea.gov.sg (K.W.M.); Wong_Wing_yan@nea.gov.sg (W.Y.W.); 2 Tan Tock Seng Hospital, 11 Jalan Tan Tock Seng, Singapore 308433, Singapore; Yee_Sin_Leo@ttsh.com.sg; 3 CMC Wong Binjai Clinic, 15 Binjai Park, Singapore 589824, Singapore; cmcWongBinjaiClinic@hotmail.sg; 4 School of Biological Sciences, Nanyang Technological University, Singapore 637551, Singapore

**Keywords:** Zika virus, antibody, mosquito

## Abstract

We report a case of a Singaporean who acquired Zika virus (ZIKV) during a visit to Cuba. The infection was confirmed using molecular and serological methods. This report highlights potential drawbacks of using IgG serology for diagnosis of flavivirus infections in endemic regions. The low viremia detected during the early phase of this case resulted in low mosquito infectivity rates, suggesting the possibility of ZIKV transmission prior to clinical onset. The report also emphasizes the challenges of public health interventions for Zika fever and the importance of sustaining a low vector population to reduce the risk of arbovirus transmission in vulnerable regions.

## 1. Introduction

Zika virus (ZIKV) caused a major outbreak in the Pacific Island of Yap in 2007 [1]. Global spread brought the virus to the Americas, where it has been associated with neurological, developmental, and ophthalmological abnormalities [2,3]. For the past four decades, Zika virus has been reported in South East Asia—including Malaysia in 1969 and Indonesia in 1977 [4,5]. However, it attracted little attention until the huge outbreaks in the Americas [6] and since then, several countries had reported autochthonous transmission of ZIKV [7]. This included Singapore where transmission, limited to a single lineage of the Asian genotype, has been documented in Singapore since August 2016 [8]—458 cases in 2016 and 67 cases in 2017 [9]. ZIKV is primarily transmitted by the *Aedes* species of mosquitoes, which also transmits the Dengue virus (DENV) [10]. Because the two closely related flaviviruses overlap greatly in geographical spread [11], and the serological tests of the two viruses inter-cross react [12], ZIKV diagnosis using serology remains a challenge in endemic settings [1,13].

## 2. The Study

**Figure 1:**
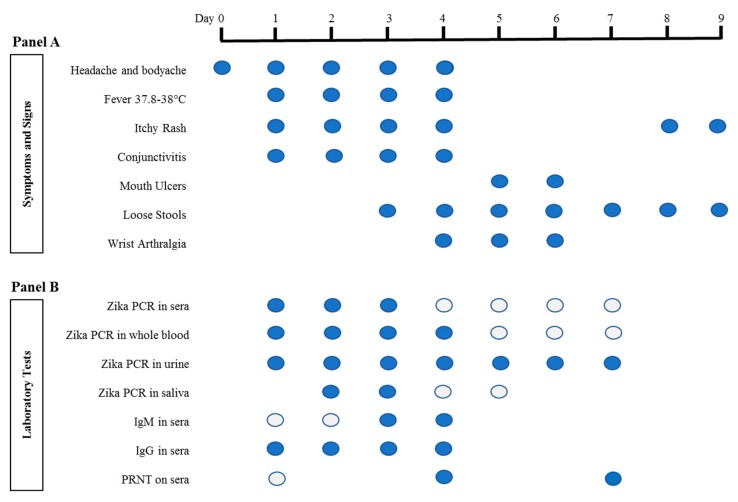
Summary of symptoms, signs, and laboratory test results. For Panel A, 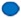 represents the presence of symptoms and signs on any particular day. For Panel B laboratory tests, each circle represents a sample that was tested. 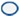 represents negative, while 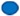 represents positive results. Saliva was collected as previously described [14]. IgM and IgG serology was performed with Zika SD Biosensor rapid kit (SD Biosensor, Gyeonggi-do, Republic of Korea) according to the manufacturer’s instructions. RT-quantitative PCR (qPCR) was performed as previously reported [1]. RNA was extracted from sera, urine and saliva using QIAamp© Viral RNA Mini Kit (Qiagen, Hilden, Germany), and from whole blood using High Pure Viral Nucleic Acid Kit (Roche Diagnostics, Mannheim, Germany) according to the manufacturers’ instructions. Zika Virus (ZIKV) PRNT was performed as previously described [15], except that ZIKV was used instead of Dengue virus. PRNT: Plaque Reduction Neutralization Test; PCR: Polymerase Chain Reaction.

**Figure 2:**
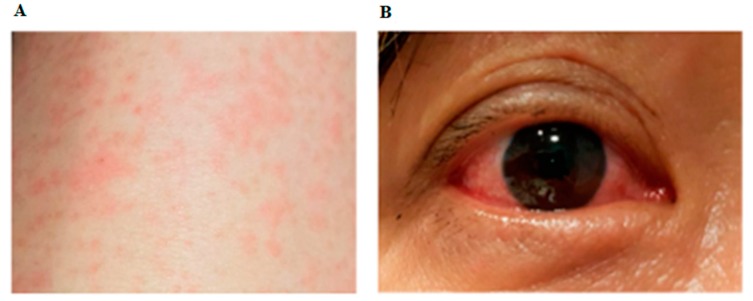
(**A**,**B**) Clinical images. (**A**) Maculopapular rash on arm; (**B**) Non-purulent conjunctivitis.

In August 2017, a 51-year old female infected with the American strain of Asian genotype (NCBI accession No. MF988734) was reported in Singapore. The patient left Singapore for Havana, Cuba on 6 August 2017, and returned on 13 August, one day before the onset of symptoms. She initially presented with headache and neck/shoulder ache (day 0) and became febrile (37.8–38 °C) on the following day (Figure 1). Fever was associated with an itchy maculopapular rash and mild conjunctivitis (Figure 2A,B). The rash initiated from the arms and back of the torso, symmetrically disseminated to the face and whole body (including soles, palms and between fingers and toes) by day 3–4, and subsided on day 5. The rash reappeared on day 8 and lasted for two days. She developed bilateral numbness of thumb, index and middle fingers on day 4 and swelling of the wrist and hand from day 4 to 6. Despite uneventful recovery on day 9, the patient continued to experience skin tenderness when touched (tactile allodynia) for two days. Case had loose stool, once or twice a day from day 3 to 9. Fever and body aches were relieved by the antipyretics and analgesics.

Laboratory tests conducted on day 4 of illness showed leukopenia (3.1 × 10^9^/L) and reactive lymphocytosis (0.28 × 10^9^/L). A nerve conductance test on day 5 provided no evidence of diffuse peripheral neuropathy, with motor, sensory and late responses of the upper and lower limbs within normal ranges. Guillain-Barre Syndrome (GBS) was ruled out, and the numbness of the fingers was probably due to transient compression of the median nerve as a result of wrist joint swelling.

ZIKV infection was confirmed by RT-qPCR [1] on the urine sample collected on day 1 of illness. ZIKV RNA (4.59–6.69 log_10_RNA copy/mL) remained detectable in all urine samples (*n* = 15) up to day 7 and RNA titres had no correlation with the hour of urine collection. Viral RNA was detectable up to day 6 in whole blood, but up to day 3 in sera. Day 1 sera yielded the highest viremia (7.49 log_10_RNA copy/mL; 50 pfu/mL by plaque assay), which dropped on day 2 to 6.03 log_10_RNA copy/mL and day 3 to 5.02 log_10_RNA copy/mL. ZIKV RNA was also detectable in day 2–3 saliva samples. Virus was isolated from urine and sera samples using *Aedes albopictus* cell line (C6/36, ATCC© CRL-1660™) for at least three passages, and confirmed by immunofluorescence assay with flavivirus group antigen complex (ATCC© HB112™) and sequencing. No cytopathic effect was observed in the infected cell cultures.

IgM was first detected on day 3, and IgG on day 1. Patient had previously tested positive for Dengue virus (DENV)-1 and -2 neutralising antibodies by Plaque Reduction Neutralisation Test (PRNT_50_) assay [15], and had vaccination against Yellow Fever and Japanese encephalitis viruses in 2008 and 2012 respectively. The early emergence of ZIKV-reactive IgG was likely due to the stimulation of cross-reactive antibodies from past flavivirus exposures—similar to the circumstances surrounding a secondary dengue infection. PRNT_50_, adapted from Low et al. [15], using three strains of ZIKV (Uganda strain, MR 766, ATCC VR-84; Thailand strain, NCBI accession No. KF993678; Puerto Rico strain, PRVABC59, ATCC VR-1843) showed an increasing titre from <10 on day 1, 32–90 on day 4 to 180–450 on day 8. Case was negative for dengue (Non-structural 1 (NS1) antigen and IgM) and chikungunya (PCR and IgM).

**Figure 3:**
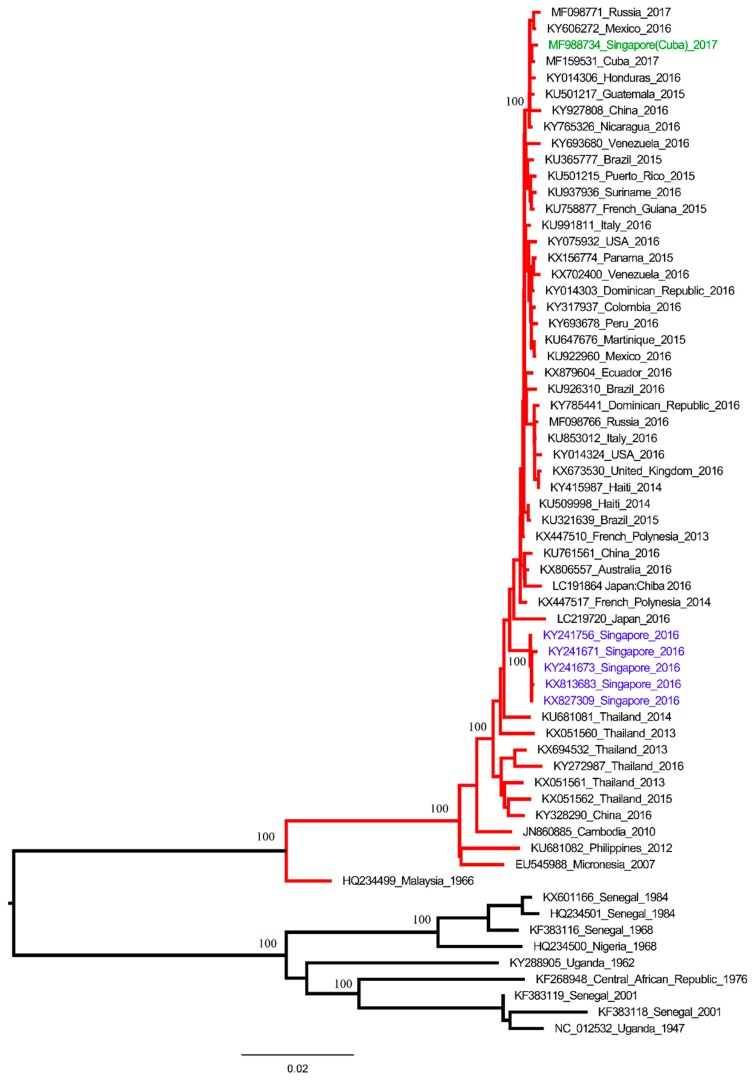
Phylogeny of ZIKV complete polyprotein. The case sequence is highlighted in green, whereas the outbreak strains in Singapore are highlighted in blue. Phylogenetic analysis was performed in MEGA7 (http://megasoftware.net/) program using the maximum-likelihood method based on the general time reversible model with gamma distribution and invariant sites. The robustness of the original tree was tested with 1000 bootstrap replications. Asian and African genotypes are represented by red and black branches respectively. Numbers on branches are bootstrap support values.

The complete genome sequence of the isolate shared the highest nucleotide (99.8%) and amino acid (100%) similarity with a Cuban strain (NCBI accession No. MF159531) detected in the United States of America in April 2017. The virus was distinct from autochthonous strains circulating in Singapore since August 2016, though both belonged to Asian genotype (Figure 3). Sequence analysis of the virus strain, along with the patient’s travel history, suggests that the infection occurred in Cuba. ZIKV whole genome was sequenced as per the protocol described in Supplementary File S1.

Day 1 to 4 residual ethylenediaminetetraacetic acid (EDTA) blood samples were fed to local *Aedes aegypti* mosquitoes (*n* = 100) [16], which were then sacrificed to test for ZIKV (Table 1). Viable ZIKV was recovered from only one midgut of 30 mosquitoes fed with day 1 blood sample. None of the mosquitoes yielded viable ZIKV in the salivary gland, suggesting non-infectivity. From each feed, ZIKV RNA was detected only in 10–20% of mosquito heads, showing low level of viral dissemination. The low infectivity of ZIKV was in contrast to DENV, in which the viremia of ~7 log_10_RNA copy/mL resulted in 90–100% of midgut and 25–50% of salivary gland infection rates [16]. To the best of our knowledge, this is the first report using a patient’s venous EDTA blood to infect mosquitoes. Unfortunately, the ZIKV titre in the EDTA blood was low and could have resulted in the low mosquito infection rate. The low infectivity also greatly differed from the high infection rate (as high as 100%) reported earlier based on laboratory studies that used high ZIKV titres to feed mosquitoes [17,18]. However, it has been shown that the susceptibility of geographically different populations of *Ae. aegypti* mosquitoes vary among different ZIKV strains [19,20,21]. Pompon et al. [17] has also reported the absence of infection among local *Ae. aegypti* mosquitoes fed with low ZIKV titres (10^2^ pfu/mL).

## 3. Conclusions

The case exhibited classical symptoms of Zika infection [22], with no evidence of GBS. Distinct virus lineages circulating in Asia and the Americas suggest that outbreaks on the two sides of the globe are independent, and possibly driven by environmental factors such as the El Nino of 2015–2016 [23]. This report highlights the limit of IgG serology against the backdrop of flavivirus endemicity. It also demonstrates the risk of cross border exchanges of emerging viruses. The low viremia and low mosquito infectivity rate, as early as day 2 of disease, and their subsequent plunge suggest that the case was probably more infective before the clinical onset. Together with the high rate of asymptomatic cases, these factors contribute to the challenges of public health interventions for Zika fever and highlight the importance of sustaining a low vector population to reduce the risk of arbovirus transmission in vulnerable regions.

## Figures and Tables

**Table 1:** Infection of *Aedes aegypti* (F2 generation).

Days Post Infection	Fever Days (Zika virus log_10_RNA Copy/mL)/Infection Rate (*n*)											
	Day 1 (7.49)			Day 2 (6.03)			Day 3 (5.02)			Day 4 (Undetectable)		
	MG	SG	Head	MG	SG	Head	MG	SG	Head	MG	SG	Head
5	10% * (10)	0 (10)	10% (10)	0 (10)	0 (10)	20% (10)	ND			ND		
7	0 (10)	0 (10)	10% (10)	0 (10)	0 (10)	0 (10)	0 (10)	0 (10)	10% (10)	0 (10)	0 (10)	10% (10)
14	0 (10)	0 (10)	10% (10)	0 (10)	0 (10)	20% (10)	0 (10)	0 (10)	10% (10)	0 (10)	0 (10)	0 (10)

Female mosquitoes were fed with residual EDTA blood samples (drawn within 2 h). Fully engorged mosquitoes were incubated at 28 °C ± 1 °C with 80% ± 10% relative humidity. Midgut (MG) and salivary glands (SG) were dissected at 5, 7 and 14 days post infection and viral titres were determined using 50% tissue culture infectious dose assay (log_10_ TCID_50_/mL) [18]. Mosquito heads were analyzed for ZIKV RNA using RT-qPCR as previously described [1]. The discrepancy in the results between the cell-based assay and the RT-qPCR assay could be due to the higher sensitivity of the latter assay. The RT-qPCR assay measures not only the infectious virus but also non-infectious, immature and defective virions, which are not capable of further infection and replication. * 4.95 log_10_TCID_50_/mL midgut viral titre; ND = not detected.

## Supplementary Materials

Supplementary materials can be found at www.mdpi.com/1999-4915/10/1/44/s1.

## References

[1] Lanciotti R.S., Kosoy O.L., Laven J.J., Velez J.O., Lambert A.J., Johnson A.J., Stanfield S.M., Duffy M.R. (2008). Genetic and serologic properties of zika virus associated with an epidemic, Yap State, Micronesia, 2007. Emerg. Infect. Dis..

[2] Krauer F., Riesen M., Reveiz L., Oladapo O.T., Martinez-Vega R., Porgo T.V., Haefliger A., Broutet N.J., Low N., WHO Zika Causality Working Group (2017). Zika virus infection as a cause of congenital brain abnormalities and guillain-barre syndrome: Systematic review. PLoS Med..

[3] Heukelbach J., Alencar C.H., Kelvin A.A., de Oliveira W.K., Pamplona de Góes Cavalcanti L. (2016). Zika virus outbreak in Brazil. J. Infect. Dev. Ctries..

[4] Marchette N.J., Garcia R., Rudnick A. (1969). Isolation of zika virus from *Aedes aegypti* mosquitoes in Malaysia. Am. J. Trop. Med. Hyg..

[5] Olson J.G., Ksiazek T.G., Suhandiman, Triwibowo (1981). Zika virus, a cause of fever in Central Java, Indonesia. Trans. R. Soc. Trop. Med. Hyg..

[6] Ikejezie J., Shapiro C.N., Kim J., Chiu M., Almiron M., Ugarte C., Espinal M.A., Aldighieri S. (2017). Zika virus transmission—Region of the Americas, May 15, 2015–December 15, 2016. Morb. Mortal. Wkly. Rep..

[7] Hills S.L., Fischer M., Petersen L.R. (2017). Epidemiology of zika virus infection. J. Infect. Dis..

[8] Singapore Zika Study Group (2017). Outbreak of zika virus infection in Singapore: An epidemiological, entomological, virological, and clinical analysis. Lancet Infect. Dis..

[9] Ministry of Health Weekly Infectious Disease Bulletin. https://www.moh.gov.sg/content/dam/moh_web/Statistics/Infectious_Diseases_Bulletin/2017/December/2017_week_52.pdf..

[10] Gardner L., Chen N., Sarkar S. (2017). Vector status of *Aedes* species determines geographical risk of autochthonous zika virus establishment. PLoS Negl. Trop. Dis..

[11] Bhatt S., Gething P.W., Brady O.J., Messina J.P., Farlow A.W., Moyes C.L., Drake J.M., Brownstein J.S., Hoen A.G., Sankoh O. (2013). The global distribution and burden of dengue. Nature.

[12] Priyamvada L., Quicke K.M., Hudson W.H., Onlamoon N., Sewatanon J., Edupuganti S., Pattanapanyasat K., Chokephaibulkit K., Mulligan M.J., Wilson P.C. (2016). Human antibody responses after dengue virus infection are highly cross-reactive to zika virus. Proc. Natl. Acad. Sci. USA.

[13] Dasgupta S., Reagan-Steiner S., Goodenough D., Russell K., Tanner M., Lewis L., Petersen E.E., Powers A.M., Kniss K., Meaney-Delman D. (2016). Patterns in zika virus testing and infection, by report of symptoms and pregnancy status—United States, January 3–March 5, 2016. Morb. Mortal. Wkly. Rep..

[14] Yap G., Sil B.K., Ng L.C. (2011). Use of saliva for early dengue diagnosis. PLoS Negl. Trop. Dis..

[15] Low S.L., Lam S., Wong W.Y., Teo D., Ng L.C., Tan L.K. (2015). Dengue seroprevalence of healthy adults in singapore: Serosurvey among blood donors, 2009. Am. J. Trop. Med. Hyg..

[16] Tan C.H., Wong P.S., Li M.Z., Yang H.T., Chong C.S., Lee L.K., Yuan S., Leo Y.S., Ng L.C., Lye D.C. (2016). Membrane feeding of dengue patient’s blood as a substitute for direct skin feeding in studying aedes-dengue virus interaction. Parasites Vectors.

[17] Pompon J., Morales-Vargas R., Manuel M., Huat Tan C., Vial T., Hao Tan J., Sessions O.M., Vasconcelos P.D.C., Ng L.C., Missé D. (2017). A zika virus from america is more efficiently transmitted than an asian virus by *Aedes aegypti* mosquitoes from Asia. Sci. Rep..

[18] Li M.I., Wong P.S., Ng L.C., Tan C.H. (2012). Oral susceptibility of Singapore *Aedes* (*Stegomyia*) *aegypti* (*Linnaeus*) to zika virus. PLoS Negl. Trop. Dis..

[19] Roundy C.M., Azar S.R., Rossi S.L., Huang J.H., Leal G., Yun R., Fernandez-Salas I., Vitek C.J., Paploski I.A., Kitron U. (2017). Variation in *Aedes aegypti* mosquito competence for zika virus transmission. Emerg. Infect. Dis..

[20] Ciota A.T., Bialosuknia S.M., Ehrbar D.J., Kramer L.D. (2017). Vertical transmission of zika virus by *Aedes aegypti* and *Ae. Albopictus* mosquitoes. Emerg. Infect. Dis..

[21] Chouin-Carneiro T., Vega-Rua A., Vazeille M., Yebakima A., Girod R., Goindin D., Dupont-Rouzeyrol M., Lourenço-de-Oliveira R., Failloux A.B. (2016). Differential susceptibilities of *Aedes aegypti* and *Aedes albopictus* from the Americas to zika virus. PLoS Negl. Trop. Dis..

[22] Mittal R., Nguyen D., Debs L.H., Patel A.P., Liu G., Jhaveri V.M., S Kay S.I., Mittal J., Bandstra E.S., Younis R.T. (2017). Zika virus: An emerging global health threat. Front. Cell. Infect. Microbiol..

[23] Caminade C., Turner J., Metelmann S., Hesson J.C., Blagrove M.S., Solomon T., Morse A.P., Baylis M. (2017). Global risk model for vector-borne transmission of zika virus reveals the role of el niño 2015. Proc. Natl. Acad. Sci. USA.

