# Supplementary material for: Viral and Antibody Kinetics, and Mosquito Infectivity of an Imported Case of Zika Fever Due to Asian Genotype (American Strain) in Singapore
Source: Viruses. 2018 Jan 18;10(1):44. doi: 10.3390/v10010044 (PMC5795457; doi:10.3390/v10010044)
Supplement: Supplementary file 1 [file viruses-10-00044-s001.pdf]

Supplementary S1: Protocol for ZIKV whole genome sequencing.

Viral RNA extraction and complementary DNA synthesis

Viral RNA was extracted from culture supernatants using the QIAGEN QIAamp viral RNA minikit (QIAGEN, Hilden, Germany) according to the manufacturer's recommendations. The complementary DNA (cDNA) was synthesised by using random hexamers and 5 µl of RNA according to the Maxima H Minus First Strand cDNA Synthesis Kit (Thermo Fisher Scientific, USA) protocol recommended by the manufacturer.

ZIKV whole genome sequencing

Amplification and sequencing primers were designed to cover the entire genome of ZIKV in overlapping fragments. Additional two sets of primers were used to capture 5' and 3' untranslated regions (UTRs). The primer sequences are given in tables below. Each genome fragment was amplified by PCR using 0.5 µM of each primer, 2 µl of cDNA and 1X Phusion™ Flash High-Fidelity PCR Master Mix (Thermo Fisher Scientific, USA). The amplification protocol is as follows: initial denaturation at 98°C for 10 sec, 35 cycles of denaturation at 98°C for 5 sec, annealing at 64°C for 10 sec, extension at 72°C for 45 sec and final extension at 72°C for 2 min. Amplified products were visualized in 2% agarose gels stained with GelRed (Biotium Inc., USA). Amplified products were purified using Expin PCR SV mini kit (GeneAll Biotechnology, Korea) according to manufacturer's instructions. Sequencing of purified PCR products was performed at a commercial sequencing facility according to the BigDye Terminator Cycle Sequencing kit (Applied Biosystems, USA) protocol. Raw nucleotide sequences were assembled using the Lasergene package version 8.0 (DNASTAR Inc., Madison, WI, USA) to obtain the consensus sequence.

Primers used for the amplification of genome fragments

Overlapping Fragments	Primer	Sequence (5′ – 3′)	Product size (bp)
F1	ZIKA 47F	CGARAGCTARCAACAGTATCAACAG	2541
	ZIKA 2587R	TGTCCCTCCAGGCTTCAAC	
F2	ZIKA 2207F	CATTTGARGCCACTGTGAGAG	2020
	ZIKA 4226R	TCCGCTTCCCACTCCTTGT	
F3	ZIKA 4042F	CTGCTTGTGGCRTGGAGAG	2026
	ZIKA 6067R	TCTTGCTTCAAGCCAGTGTGC	

F4	ZIKA 5737F	GYTGTCTGACAAAGGCTGGAA	1999
	ZIKA 7735R	CAGGGCCGACATCTGRTTC	
F5	ZIKA 7561F	GAACTCCTCTACAGCCACYTCACT	2163
	ZIKA 9723R	TCCCATRTCATTCAAGAACCTGA	
F6	ZIKA 9302F	AGGTTTGATCTGGAGAATGAAGC	1452
	ZIKA 10753R	CAGCGTGGTGGAAACTCAT	

Primers used for the amplification of 5′UTR and 3′UTR regions

Overlapping Fragments	Primer	Sequence (5′ – 3′)	Product size (bp)
5′UTR	ZIKV_5UTRF1	GTAAAACGACGGCCAGTTGTTGA TCTGTGTGARTC	161
	ZIKV_5UTRF2	GTAAAACGACGGCCAGTTGTTAC TGTTGCTGACTC	
	ZIKV_5UTRR	TTTAGCATATTGACAATCCG	
3′UTR	ZIKV_3UTRF	CTAGTCAGCCACAGYTTG	406
	ZIKV_3UTRR1	GTAAAACGACGGCCAGTAAGAC CCATGGATTTCC	
	ZIKV_3UTRR2	GTAAAACGACGGCCAGTAGAAA CCATGGATTTCC	

Primers used for the sequencing of each genome fragment

Overlapping Fragments	Primer	Sequence (5′ – 3′)
F1	ZIKA_47F	CGARAGCTARCAACAGTATCAACAG
	ZIKA_686R	GTGTTGCACCAGYAATCGAC
	ZIKA_699F	TTGGGTTGTGTACGGAACCTG
	ZIKA_1250R	TGAGTGTCTGATTGCTTGTCAAGG
	ZIKA_1311F	CAAAGGGAGCYTGGTGACATG
	ZIKA_1808F	AGGCTGAGATGGATGGTGCA
	ZIKA_1703R	TTGTTGTTCCAGTGTGGAGTTCC
	ZIKA_2587R	TGTCCCTCCAGGCTTCAAC
F2	ZIKA_2207F	CATTTGARGCCACTGTGAGAG
	ZIKA_2797F	AGATTGCCMGTGCCTGTGA
	ZIKA_3375F	ACCATCTCTGAGATCAACYACTGC
	ZIKA_3132R	CCTCTTCAGCCTCCATGTGTC

	ZIKA_3770R	GCYACATCTCCTCCAGTGTTCA
	ZIKA_4226R	TCCGCTTCCCACTCCTTGT
F3	ZIKA_4042F	CTGCTTGTGGCRTGGAGAG
	ZIKA_4541F	CTGTGGCATGAACCCAATAGC
	ZIKA_5227F	CTTCCTGAAATAGTCCGTGAAGC
	ZIKA_4779R	TCCTTTTGTRACGTGCCACAT
	ZIKA_5414R	GTGAARGTGGCATGGCACAT
	ZIKA_6067R	TCTTGCTTCAAGCCAGTGTGC
F4	ZIKA_5737F	GYTGTCTGACAAAGGCTGGAA
	ZIKA_6289F	AGAAGATGGTGCTTTGATGGC
	ZIKA_6830F	AGCCAGARAAGCAAAGATCTCC
	ZIKA_6635R	AGCATRATGGTCTCTAGGGTCTCC
	ZIKA_7148R	ACARCACTCCAGCTTGYGTG
	ZIKA_7735R	CAGGGCCGACATCTGRTTC
F5	ZIKA_7561F	GAACTCCTCTACAGCCACYTCACT
	ZIKA_8115F	AGTCATCATCTAGTCCTGAAGTGGA
	ZIKA_8764F	AGAYCCCCAAGAAGGCACTC
	ZIKA_8440R	CACATCCTCCTCATATTTCACTGG
	ZIKA_9041R	TTCCCATCATGTTGTACACACA
	ZIKA_9723R	TCCCATRTCATTCAAGAACCTGA
F6	ZIKA_9302F	AGGTTTGATCTGGAGAATGAAGC
	ZIKA_9960F	GGCAGCTYCTTTATTTCCACAGA
	ZIKA_10287R	CGCACCATGTTGACTGTGT
	ZIKA_10753R	CAGCGTGGTGGAAACTCAT

Primers used for the sequencing of 5′UTR and 3′UTR regions

Fragment	Primer	Sequence (5′ – 3′)
5′UTR	Universal seq primer M13F(-20)	
	ZIKV_5UTRR	TTTAGCATATTGACAATCCG
3′UTR	Universal seq primer M13F(-20)	
	ZIKV_3UTRR1	GTAAAACGACGGCCAGTAAGACCCATGG ATTTCC

	ZIKV_3UTRR2	GTAAAACGACGGCCAGTAGAAACCATGG ATTTCC
